# Cost-effective HRMA pre-sequence typing of clone libraries; application to phage display selection

**DOI:** 10.1186/1472-6750-9-50

**Published:** 2009-05-22

**Authors:** Barry A Pepers, Menno H Schut, Rolf HAM Vossen, Gert-Jan B van Ommen, Johan T den Dunnen, Willeke MC van Roon-Mom

**Affiliations:** 1 Center for Human and Clinical Genetics, Leiden University Medical Center, Albinusdreef 2, 2333ZA Leiden, The Netherlands; 2 Department of Neurology, Leiden University Medical Center, Albinusdreef 2, 2333ZA Leiden, The Netherlands; 3 Leiden Genome Technology Center, Leiden University Medical Center, Albinusdreef 2, 2333ZA Leiden, The Netherlands

## Abstract

**Background:**

Methodologies like phage display selection, *in vitro *mutagenesis and the determination of allelic expression differences include steps where large numbers of clones need to be compared and characterised. In the current study we show that high-resolution melt curve analysis (HRMA) is a simple, cost-saving tool to quickly study clonal variation without prior nucleotide sequence knowledge.

**Results:**

HRMA results nicely matched those obtained with ELISA and compared favourably to DNA fingerprinting of restriction digested clone insert-PCR. DNA sequence analysis confirmed that HRMA-clustered clones contained identical inserts.

**Conclusion:**

Using HRMA, analysis of up to 384 samples can be done simultaneously and will take approximately 30 minutes. Clustering of clones can be largely automated using the system's software within 2 hours. Applied to the analysis of clones obtained after phage display antibody selection, HRMA facilitated a quick overview of the overall success as well as the identification of identical clones. Our approach can be used to characterize any clone set prior to sequencing, thereby reducing sequencing costs significantly.

## Background

Phage display libraries consist of small antibody fragments cloned into a display phage vector, allowing efficient antibody screening and production in a bacterial system [1,2]. Traditional antibodies are composed of a heavy- and a light-chain that need to recombine in a tetramer for the formation of a functional antibody. Because most of these random recombinations will yield non-functional antibodies, when produced as recombinant fragments in *E. coli*, isolation of effective antibodies demands extremely large phage libraries. *Camelidae *have, next to conventional antibodies, dimeric heavy chain antibodies (HCAb) that lack light chains [3]. The variable domain of the HCAb (VHH) has a single binding domain with a specificity and affinity similar to conventional antibodies [4,5]. In a phage display library, each phage displays a different antigen-binding domain on its surface. To isolate specific antibodies, phage particles from a library are bound to an antigen, recovered and used to infect fresh bacteria. Subsequently, phages go through several rounds of epitope binding and re-infection resulting in an enrichment of binding phages. A perfect experiment will ultimately yield groups of phages, each encoding a different antibody directed against the starting antigen. The set of phages can be used together as 'polyclonal phages', individual phages as 'monoclonal phages'. After selection, individual VHH clones are characterized to determine their specificity by ELISA and their diversity by fingerprinting/sequencing. Although ultimate identification is done using clone-insert nucleotide sequencing, pre-sequence fingerprinting is performed to reduce cost. Phage display clones are usually analysed using restriction digestion of PCR amplified VHH insert, followed by agarose gel-electrophoresis [4]. However, this methodology is time consuming, labour intensive, has limited resolution and is not effective for the analysis of a large number of clones.

In the current study, we developed a protocol using high resolution melt curve analysis (HRMA) to visualise clonal diversity and study enrichment of clones after VHH-selection from a llama non-immune phage display library. Unlike the traditional application for melt curve analysis, where 1 base pair differences are detected through a change in melt temperature of a fully base-paired hybrid and mismatched hybrids, the current study uses differences in melt curve shape and the Tm of each melt curve to identify template nucleotide sequence similarities within a large group of unlike PCR fragments. Similar melt curve shapes represent similar DNA sequences and melt curves can be automatically and efficiently grouped using the available HRMA software.

## Results

**Figure 1:**
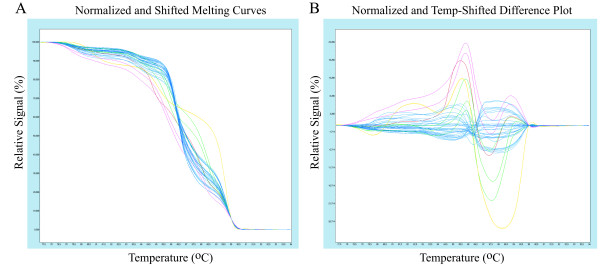
**Grouping of clones by high resolution melt curve analysis (HRMA)**. A) Normalized and temperature shifted melt curves for the 25 samples that showed a positive result with ELISA. Samples with differences in their DNA sequences can be easily distinguished because of their different shaped melt curves. The different groups as assigned by HRMA are represented by different colours. B) Shows the same samples as in A. The differences in melt curve shapes are further analysed by using the sample with the highest melting temperature from the normalized and temperature shifted melt curves as a baseline in order to cluster samples automatically into groups that have similar melt curves. The different groups as assigned by HRMA are represented by different colours.

**Figure 2:**
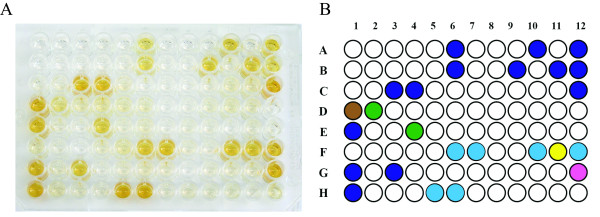
**Comparison of ELISA results and high resolution melt curve analysis (HRMA)**. A) ELISA results for the 96 colonies selected from the phage display library. B) The different colours represent the different groups as assigned by HRMA. Only results for the ELISA positive clones are shown.

**Figure 3:**
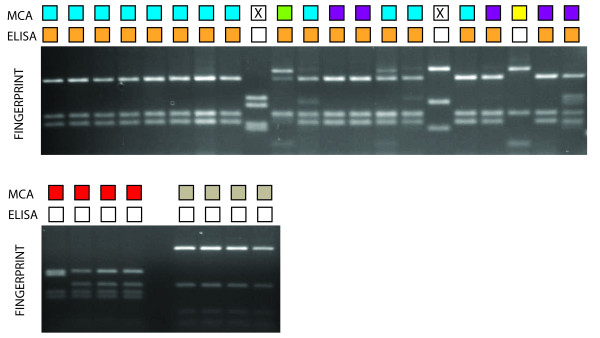
**Comparison of ELISA results, high resolution melt curve analysis (HRMA) and restriction digestion analysis**. Shown in the top row of the small square boxes are representative clones from groups as assigned by HRMA. The different colours indicate the different groups, and a cross indicates these samples could not be analysed because of a low quality melt curve. The second row of boxes indicates the ELISA results. A white box represents a negative ELISA result and an orange box indicates a positive ELISA result. The corresponding restriction digestion pattern for each sample is shown in the lower panel. HRMA identifies an additional group in the ELISA-positive clones (top panel sample 12,13, 18, 20 and 21) not identified by fingerprint analysis.

After two rounds of selection against an epitope spanning the first 548 amino acids of the huntingtin protein [6], 96 phages were picked and ELISA showed 25 positive and 71 negative wells. An optical density of 0.6 or higher was considered a positive result while the negative control was less than 0.1. Clone diversity was investigated using both HRMA and *Hin*fI restriction digestion of PCR-amplified clone inserts. As expected, since the PCR fragments had an average size of 600 bp, HRMA showed a wide range of melt profiles often containing multiple melting domains per fragment representing differences in nucleotide sequence. Representative results from 4 independent HRMA analyses are shown in Figure 1, a comparison of the ELISA and HRMA results are shown in Figure 2. Only the ELISA-positive clones are represented in this figure. There was a complete agreement of ELISA-positive and ELISA-negative clones with HRMA analysis. The 25 ELISA-positive clones belonged to 6 different groups, the largest group contained 14 clones, one group 6 clones, one group 2 clones, and 3 groups contained a unique clone. Of the remaining 71 clones that showed a negative ELISA, 12 clones could not be analysed by HRMA because of a low PCR yield and/or low quality melting. The remaining 59 clones could be grouped into 20 different groups, consisting of one large group of 17 clones and three groups of 8, 6, and 4 clones, respectively. The remaining groups all contained 3 clones or less. All groups identified by restriction digestion fingerprinting were also identified by HRMA. Groups could overall be confirmed by nucleotide sequence analysis and PCR fragments were approximately 600 bp in length ranging from 552 to 607 bp with a Tm of either 85 or 86°C and a G/C content of 53 or 54%. The most common fragment length was 606 bp with a Tm of 86°C and a G/C content of 54%. Fragments with these characteristics were present in the majority of groups assigned by the software. As can be seen in Figure 3, HRMA even identified one additional group that was not seen with fingerprinting. However, as can be seen in the red HRMA group in Figure 3, occasionally, ELISA results and HRMA results did not agree with restriction digestion results. Sequencing showed that the red clone with the different digestion pattern was 2 bp different from the other clones in this HRMA group. These changes were outside the VHH encoding region and thus did not affect VHH binding characteristics. However, this 2 bp difference did change a *Hin*fI restriction site resulting in a different restriction pattern.

## Discussion

HRMA has been used routinely for mutation scanning on patient samples with small fragments of equal size (up to 400 bp) that differ only 1 or a few nucleotides in sequence [7]. The temperature at which the probe-template hybrid melts, distinguishes between fully base-paired hybrids from mismatched hybrids. In the current study, we developed a protocol using HRMA to identify clonal origin of VHH fragments selected from a phage display library as an alternative for DNA fingerprinting. The protocol uses the differences in melt curve shape and Tm of each melt curve to identify template nucleotide sequence similarities within a large group of samples. Although the software used for HRMA was originally designed to detect 1 bp differences between two small fragments, our results demonstrate that it is also capable of accurately analysing melt curves from longer unlike PCR fragments. HRMA is a very efficient technique to obtain a quick overview and determine if clone selection was successful. From a successful selection experiment one expects several recurring clones. When all clones are different, no enrichment has taken place and clone selection has probably failed, when all clones are identical, the success is probably also doubtful. When the ELISA negative clones contain a large recurrent set, these clones might either be well-growing but have low-affinity/are non-specific (not interesting) or high affinity clones that somehow fail to produce enough VHH in order to obtain a positive ELISA signal (interesting). HRMA is also suitable to follow clone selection; initially one expects all clones to be different, with larger clone sets emerging in later rounds of selection. When comparing HRMA to digestion analysis of the VHH fragments, HRMA analysis is a more sensitive and efficient method to determine clonal similarity. All groups identified with restriction digestion fingerprinting could also be identified by HRMA and were overall confirmed by sequence analysis. It is a simple and rapid method taking only two and a half hours to complete after PCR amplification. Furthermore, it is inexpensive, requiring only PCR, a DNA dye, and melting instrumentation. In the current study we have used the LightCycler480 for the final PCR amplification and melt curve analysis, however, any block cycler can be used for amplification and the subsequent melt analysis could then be performed with other high-resolution melting devices such as the LightScanner (Idaho Technology Inc, Salt Lake City, Utah). Multiple samples can be analysed simultaneously (HRMA facilitates analysis of up to 384 clones per microtitre plate) and groups can be assigned in an automated manner.

## Conclusion

HRMA is very efficient to obtain a quick overview and determine if clone selection was successful. Similar groups of clones identified by restriction digestion fingerprinting were also identified by HRMA and were confirmed by nucleotide sequence analysis. HRMA is suitable for a wide variety of applications where verification and/or analysis of clonal diversity is essential, including determining clone diversity in a single-chain (sc) Fv phage library [8], analysis of clones obtained after *in vitro *mutagenesis [9], cDNA clones to determine allelic expression [10] and clones to determine methylation status of genomic regions [11].

## Methods

### ELISA and restriction digestion

Two consecutive rounds of selection were performed with a large non-immune VHH library as described previously [4,5] on the first 548 amino acids of the huntingtin protein [6]. From this selection, 96 individual colonies were randomly picked. To assess specific binding to the antigen of interest, ELISA was performed. In short, all 96 clones were grown first in 100 μl/well of growth medium (2xTY/Ampiciline/0,1% Glucose) for 4 hours at 37°C while shaking (220 rpm). Induction of VHH overproduction was then performed overnight after adding 20 μl of a 6 mM IPTG solution (in 2xTY/Ampiciline). A 96 well Maxisorp plate was coated with antigen overnight at 4°C. The next day; Maxisorp plates were washed twice with 1× PBS and blocked for 30 min at room temperature with 4% non-fat milk in PBS. After blocking, 50 μl of 4% non-fat milk/PBS solution was added to each well. Plates containing the VHH's were spun down at 1200 g and 4°C for 15 minutes. From each well, 50 μl of VHH-containing supernatant was added to the corresponding well of the Maxisorp plate. The Maxisorp plate was then incubated for 2 hours at room temperature while shaking (900 rpm). After incubation the plate was rinsed 3 times with PBST and 3 times with PBS. For the detection of bound VHH's, 100 μl of a 1:1000 solution of anti-myc antibody (9E10, Santa Cruz) conjugated to Horse Radish Peroxidase (HRP) in 4% non-fat milk/PBS was added to each well. The Maxisorp plate was then incubated for 2 hours at room temperature while shaking (900 rpm). After incubation the plate was rinsed 3 times with PBS containing 0.05% Tween20 and subsequently 3 times with PBS. The reaction was developed by adding an OPD-H_2_O_2 _solution to each well followed by a 35 minutes incubation at room temperature under dark conditions. Reaction was stopped by adding 50 μl/well of 1 M H_2_SO_4_. Optical densities were measured at a wavelength of 490 nm using a plate reader (Biotek, Winooski, USA). To analyse clone inserts, PCR was performed on all 96 clones on a standard block cycler (Bio-Rad, Hercules, CA) using 1 μl of overnight culture with the following primers: M13R 5'-CAGGAAACAGCTATGAC-3' and MPE25WB 5'-TTTCTGTATGGGGTTTTGCTA-3'. Amplification was performed in 1× PCR buffer, 0.7 U FastStart Taq DNA polymerase (Roche, Mannheim, Germany), 200 μM dNTPs, 1 pmol of each primer in a reaction volume of 20 μl. Cycling conditions were 5 min at 95°C followed by 35 cycles of 40 sec at 95°C, 40 sec at 55°C and 1 min at 72°C, followed by a final incubation of 5 min at 72°C. DNA fingerprint analysis was performed on 10 μl PCR product digested for 2 hr at 37°C in a total volume of 20 μl containing, 1× reaction buffer and 1 U *Hin*fI (Fermentas, Burlington, Canada) and digests were run on a 3% agarose gel.

### High resolution melt curve analysis

Amplification for HRMA was performed on 2 μl of 1:1000 dilutions of previously amplified clones using the same primers as in the first PCR reaction in a 10 μl reaction volume containing 1× LightCycler^® ^High Resolution Melting Master (Roche), 2 mM MgCl_2_, and 3 pmol of each primer. All samples were amplified in duplicate in the Lightcyler^®^480 (Roche) and this was followed by melt curve acquisition. Initial denaturation of 10 min at 95°C was followed by 30 cycles of 10 sec at 95°C, 30 sec at 55°C and 20 sec at 72°C. After a final extension of 5 min at 72°C, melt curve acquisition started with a hold of 1 min at 95°C followed by 1 min at 40°C and ramping from 60°C to 98°C at 1°C/sec with 25 acquisitions per °C. Grouping of the clones was done using the Genescanning module of the Lightcyler^®^480 Software Release 1.5.0 (Roche). The sample with the highest melting temperature was selected from the normalized and temperature shifted melt curves and used as baseline for the difference plot analysis. After the software had calculated the groups, they were checked manually to ensure that samples with identical melt curves were assigned to their appropriate groups. Because the software could only assign 8 groups at once, analysis was done three times. For the second and third round of analysis, all samples assigned to groups in the previous rounds were omitted until all samples had been clustered.

## Competing interests

The authors declare that they have no competing interests.

## Authors' contributions

BAP helped writing the manuscript, set up HRMA, performed ELISA and restriction digestion, MHS performed ELISA, restriction digestion analysis, and sequencing, RHAMV was instrumental in developing the HRMA method, GJBO participated in the design of the study JTD critically revised the manuscript and designed the study WMCRM wrote the manuscript and supervised the study.
